# P200: No need for initial broad-spectrum empiric antibiotic coverage after surgical drainage of orthopaedic implant infections

**DOI:** 10.1186/2047-2994-2-S1-P200

**Published:** 2013-06-20

**Authors:** I Uckay, M Schindler, A Agostinho, P Hoffmeyer, D Pittet

**Affiliations:** 1 Infection Control Program, Geneva University Hospitals, Geneva, Switzerland; 2 Orthopaedic Surgery, Geneva University Hospitals, Geneva, Switzerland

## Introduction

Empiric broad-spectrum antibiotic treatment for orthopaedic implant infections after surgical lavage is common practice while awaiting microbiological results, but lacks evidence.

## Objectives

Our objective was to question the indication of broad-spectrum empiric therapy in this clinical setting.

## Methods

Single-centre cohort study conducted from 1996 to 2011. Methicillin-resistant *Staphylococcus aureus* endemicity ranged from 23-32% among clinical *S. aureus* isolates throughout the study period. Bacteremic cases were excluded.

## Results

We retrieved 342 implant infections and followed them for a median of 3.5 years (61 recurred; 18%). Infected implants were arthroplasties (n=186), different plates, nails, or other osteosyntheses. Main pathogens were *S. aureu*s (163; 49 methicillin-resistant) and coagulase-negative staphylococci (60; 45 methicillin-resistant). Median duration of empiric antibiotic coverage after surgical drainage was 3 days before switching to targeted therapy. Vancomycin was the most frequently used initial empiric agent (147), followed by intravenous co-amoxiclav (44). Most empiric antibiotic regimens (269; 79%) proved sensitive to the causative pathogen, but were too broad in 111 episodes (32%). Although they would have covered 59% of later identified causative pathogens, cephalosporins and penicillins were used only in 44 and 10 cases, respectively. Empiric anaerobic coverage was given in 130 episodes (38%), although only five co-pathogens were anaerobes. Multivariate Cox regression analysis showed that neither susceptible antibiotic coverage (compared to non-susceptible; hazard ratio, 0.7, 95% CI, 0.4-1.2) nor exaggerated broad-spectrum use (hazard ratio, 1.1, 0.8-1.5) changed remission rates.

## Conclusion

Provided that surgical drainage is performed, broad-spectrum antibiotic coverage during the first 3 days does not enhance remission of orthopaedic implant infections. If empiric agents are prescribed from the first day of infection, narrow-spectrum penicillins or cephalosporins can be considered to avoid unnecessary broad-spectrum and anti-anaerobic antibiotic use. Randomized controlled trials are needed to confirm our findings.

## Disclosure of interest

None declared

